# Effect of a Black Soldier Fly Ingredient on the Growth Performance and Disease Resistance of Juvenile Pacific White Shrimp (*Litopenaeus vannamei*)

**DOI:** 10.3390/ani11051450

**Published:** 2021-05-18

**Authors:** Andrew Richardson, João Dantas-Lima, Maxime Lefranc, Maye Walraven

**Affiliations:** 1 Innovafeed SAS, 75010 Paris, France; maxime.lefranc@innovafeed.com (M.L.); maye.walraven@innovafeed.com (M.W.); 2 IMAQUA, 9090 Lochristi, Belgium; joao.lima@imaqua.eu

**Keywords:** black soldier fly, challenge trial, growth performance, pacific white shrimp

## Abstract

**Simple Summary:**

This study investigates the use of a Black soldier fly (*Hermetia illucens*) ingredient in juvenile shrimp (*Litopenaeus vannamei*) diets at various inclusion rates (4.5, 7.5, and 10.5%), monitoring both the growth performance and then health performance in the face of three separate challenges (White spot syndrome virus, *Vibrio parahaemolyticus,* and osmotic stress). This work showed that growth performance (measured through weight gain, feed conversion ratio, and specific growth rate) of *L. vannamei* was significantly improved in a linear trend with the inclusion of the Black soldier fly ingredient (*p* < 0.05), whilst health performance was not significantly altered. Overall, the Black soldier fly ingredient proves to be a promising additive for *L. vannamei* diets, impacting performance and sustainability positively.

**Abstract:**

This study was performed as part of developing a functional feed ingredient for juvenile Pacific white shrimp (*Litopenaeus vannamei*). Here we assess the effects of dietary inclusion of a Black Soldier Fly Ingredient (BSFI) from defatted black soldier fly (*Hermetia illucens*) larvae meal on growth performance, tolerance to salinity stress, and disease resistance when challenged with *Vibrio parahaemolyticus* or a strain of white spot syndrome virus (WSSV). A control diet was used for comparison with three test diets including 4.5, 7.5, and 10.5% of BSFI (BSFI4.5, BSFI7.5, and BSFI10.5). After 28 days, all diets with BSFI had improved weight gain, feed conversion ratio (FCR) and specific growth rate (SGR) compared to control. Indeed, SGR was significantly improved from inclusion of 4.5% in the diet, whilst FCR was significantly improved at 7.5% (*p* < 0.05). During the growth trial, survival was not affected by diet. Shrimp health performance was not significantly affected by the diets across the disease and salinity challenges. Overall, the results indicate that the inclusion of BSFI from *H. illucens* improves the performance of juvenile *L. vannamei*.

## 1. Introduction

Plant-based ingredients have become, in the past decades, common ingredients within aquafeeds [1,2,3]. However, plant-based proteins present some nutritional limitations for aquafeed: the presence of anti-nutritional factors, high levels of fiber and non-starchy polysaccharides, inadequate fatty acids, unbalanced essential amino acid profiles, and lower digestibility and palatability [2,4,5,6,7]. In addition, complete substitution of fishmeal by plant ingredients could cause additional pressures on essential agricultural resources, through a notable increase in freshwater, land, and phosphorus usage [8]. This has driven research efforts for higher performing alternative ingredients, such as byproducts of terrestrial biomass, microbial biomass, algae, and insects [3,9].

Pacific white shrimp (*Litopenaeus vannamei*) is the most produced shrimp species worldwide, with production levels surpassing 4.96 million metric tons in 2018 [10]. To support the industry’s growth there is a need to find high-performing feed ingredients, which can be supplied sustainably and improve production systems’ efficiency to deliver larger volumes while putting less pressure on natural resources.

In 2017, regulatory changes within the European Union (EU) allowed for the inclusion of insect derived ingredients in aquafeeds [11], since this there has been a proliferation of research on the topic—both within the public and private sector. The past studies evaluating the inclusion of insect ingredients in aquafeeds reported contrasting growth performances among aquatic species [12,13,14,15,16]. Variance in performance of insect meal in aquafeeds may have been a product of insect rearing conditions, processing technology, diet, insect species, and stage of development (larval, pupae, nymph, or adult) [17,18]. Larvae from many insect species, including black soldier fly (*Hermetia illucens*) have been investigated for insect ingredient production [17,19,20]. The dipteran *H. illucens* is a highly promising species for aquaculture feed production, as the mass-rearing techniques for industrial production of high quality insect meal from this species are much further in their development process [21,22]. 

The amino acid profile of the insects of the order Diptera, to which the *H. illucens* belongs, has a greater level of similarity to fish meal than soy bean meal, and other insect alternatives (Orthoptera and Coleoptera) [23]. Insect meal from *H. illucens* also shows high protein content (50–70%) and has been successfully integrated into commercial aquaculture diets in recent years. Finally, the chitin content has been shown to modulate the immune system of shrimp and fish [24,25], which could result in potential health and disease resistance benefits. Resistance towards acute hepatopancreatic necrosis disease (AHPND) and white spot syndrome virus (WSSV) are of great interest, given their role in major mortality outbreaks that have been affecting shrimp production worldwide [26,27].

When it comes to shrimp feed, the only study thus far analyzing the introduction of *H. illucens* ingredients has reported no effects on growth performance [16] although there are works exploring other species [14,28]. The present study aimed to evaluate the effect of dietary integration of a new ingredient derived from *H. illucens* larvae on juvenile *L. vannamei* growth performance, tolerance to salinity stress, and resistance to WSSV and *Vibrio parahaemolyticus* (the causative agent of AHPND).

## 2. Materials and Methods

### 2.1. Experimental Diets

The experimental ingredient (referred to as BSFI) consists of an *H. illucens* meal with a high crude protein content (60% CP) of commercial origin (InnovaFeed, Paris, France). The BSFI, in addition, contained ~4% chitin and ~7% lipid (of which ~40% is lauric acid–C12). The BSFI was obtained from larvae which were harvested before the prepupal stage, killed by heat shock, dried and defatted mechanically (no solvent was used). It is noted the *H. illucens* larvae were reared on Food Grade GMP+ certificated substrate consisting of wheat bran and cereal mill offal.

Four experimental diets were formulated and produced at IMAQUA, Aquaculture Immunology Technologies (Lochristi, Belgium) to meet the nutritional requirements of *L. vannamei* (Table 1). A high quality 15% fish meal diet was used as control and three test diets were formulated to include 4.5, 7.5, and 10.5% of the BSFI (BSFI4.5, BSFI7.5, and BSFI10.5) produced by InnovaFeed, replacing fishmeal at a 1:1 ratio. The diets were formulated to mirror practical diets used in the industry (through conversation with members of the shrimp aquafeed industry), with a total CP ~40% and fishmeal inclusion (in the case of the control) of 15%. Analytical methods used for proximate composition analysis were standard. Crude protein: Kjeldahl, from Publ EG 22/7/1993-L179/8/10; moisture: gravimetric EG-20/12/1971-L279/8/11; crude fiber: gravimetric AOAC-978.10; crude fat: gravimetric EU-L54/37-26/2/2009; ash: gravimetric ISO936. Fatty acids: gas chromatographic ISO/TS17764-2:2002. Amino acids: HPLC ISO13903:2005

All dietary ingredients were added and thoroughly mixed with a feed binder and water to pelletize the final feed. The resulting dough was pelletized with a customized heavy-duty meat grinder (2 mm die size), and strings oven dried at 50 °C for 3 h. Pellets were ground and sieved to appropriate size for 0.1 g shrimp (0.5–0.8 mm for the first 3 weeks and in last week shrimp were fed with 1.4–2 mm) and stored at 4 °C until use.

### 2.2. Shrimp Rearing and Feeding Trial

*Litopenaeus vannamei* were imported as postlarvae (PL) from Global Blue Technologies (Rockport, TX, USA). These shrimps were certified as specific pathogen free (SPF) for infectious myonecrosis, *Enterocytozoon hepatopenae*, WSSV, Taura syndrome virus, yellow-head virus, necrotizing hepatopancreatitis bacteria, infectious hypodermal and haematopoietic virus, covert mortality nordavirus, *Penaeus vannamei* nodavirus, Monodon Bacilovirus, hepatopancreatic parvovirus, AHPND/Early Mortality Syndrome (EMS), and *baculovirus penaei*. Upon arrival, shrimp PL were transported to IMAQUA facilities and reared in a water recirculation system equipped with 12 290-L plastic tanks (100 individuals in each) containing artificial seawater (salt provider Aquaculture farming technology, EX Leunen, the Netherlands) and kept at a salinity of 20 ppt. Water temperature was kept constant at 27 °C ± 1 °C by means of an automatic temperature control system, and photoperiod was 12 h light/12 h dark. During the trial, oxygen was maintained above 4 mg L^-1^ and pH ranged between 7.8 and 8.5. Water filtration was performed by biological and mechanical filters installed in each tank. No water was exchanged during the trials since water parameters were adequate throughout the experiment, in particular ammonia (NH_3_/NH_4_^+^) concentration stayed below 0.05 mg L^−1^ and nitrite (NO_2_^−^) concentration below 0.8 mg L^−1^. Ammonia, NO_2_ and KH were tested using test kits (JBL, Neuhofen Germany), and pH, temperature, and dissolved oxygen were monitored with a multimeter (WTW multi 3620 IDS, WTW, Weilheim, Germany). Salinity was measured with a digital refractometer (MA887, Milwaukee, Rocky Mount, NC, USA).

Shrimp were raised in a pre-matured RAS system on an artificial diet (replacement for live feed; MeM, Bernaqua, Belgium) and then weaned with Control and experimental diets. Each experimental diet was assigned to three groups of 100 shrimp (post larvae–PL41, 0.1 g mean initial mean body weight–MBW). For the determination of initial MBW, cohorts of 33–34 shrimp were counted and weighed in group. The groups were then distributed over the replicate tanks in such a way that the total weights were as equal as possible. Calculated variation of individual MBW was 0.0028 g. Daily survival was monitored by visually checking the tanks, any dead shrimp were removed.

Feed was automatically distributed 6 to 10 times a day over 28 days using a custom-built programable belt feeder. After this period, shrimp were randomly selected from each tank and transferred to the salinity stress test or to the disease challenge facility for the *V. parahaemolyticus* or WSSV challenging tests.

### 2.3. Growth Performance Trial

During the feeding trial, shrimp from each tank were fed the respective experimental diet. The amount of feed was calculated based on the predetermined percentages of their MBW and expected daily growth. This amount was adjusted daily according to the expected growth, observed mortality, and feed consumption per experimental group. This was also corrected after group weighing of shrimp from each treatment at days 7, 14, and 21. The following growth performance indexes were calculated at day 28 (end of the experiment): (i) weight gain (WG), (ii) specific growth rate (SGR), (iii) feed conversion ratio (FCR), and (iv) survival. FCR and SGR calculated as described by Krogdahl, et al. [29].

### 2.4. Salinity Stress Test

To assess the effect of the experimental diets on the shrimp resistance to salinity stress, 10 shrimp were randomly selected from each dietary replicate treatment after the 28 days of the growth experimental trial (salinity 20 ppt) and immediately placed directly in demineralized water (salinity 0 ppt, 27 °C) [30]. During the following 6 h, each group was monitored every 20 min. The number of dead shrimp and their time of death were recorded in each group; dead shrimp were immediately removed from the vessel.

### 2.5. Disease Challenge Tests

After the growth performance trial, shrimp were exposed to two disease challenge tests. Three days before starting the disease challenges, 260 randomly selected shrimps were transferred from the feeding trial facility to the disease challenge facility and housed individually in 10-L true experimental replicate tanks in the infection units (one shrimp per tank) for acclimatization.

For each disease challenge, three blocks of 10 shrimp from each dietary treatment (Control, BSFI4.5, BSFI7.5, and BSFI10.5) were placed at a different location of the challenge setup to account for possible variations induced by the location within the setup (120 tanks in total for each pathogen). A further 10 shrimp were housed individually for each disease challenge, these shrimps were used to produce the mock inoculum data. The challenge system comprised individual 10 L tanks equipped with their own biofilter, i.e., all tanks are true replications and independent of each other. All experimental conditions were maintained similarly to those during the shrimp rearing and feeding trials. Individual shrimp were fed manually twice daily and monitored twice daily for clinical signs of disease and mortality.

For the AHPND challenge, the *V. parahaemolyticus* strain TW01, isolated from shrimp infected with AHPND in ponds from Thailand in 2013, was characterized in the laboratory as follows: (i) it was confirmed as the causative agent of AHPND under Koch’s postulates, (ii) it was confirmed as the causative agent of AHPND by PCR using the plasmid AP2 primers, and (iii) it was confirmed as a *V. parahaemolyticus* strain using top A gene sequence analysis [31]. After thawing, the stock used in the present study was aseptically inoculated in culture medium (Müeller-Hinton, Sigma-Aldrich, St. Louis, MO, USA) and grown using standard conditions (incubation overnight at 27 °C in VWR INCU-Line 150R, on an orbital shaker Heidolph Unimax 1010 at 250 rpm). The optical density of the resulting bacterial suspension in culture medium (Müeller-Hinton, Sigma-Aldrich) was determined spectrophotometrically and based on a pre-established standard curve with a regression formula between optical density readings and plate counts. These data were used to determine the concentration of bacteria in the suspension as the number of colony forming units per milliliter (CFU/mL). Quantified suspensions of TW01 were used to inoculate shrimp by immersion in all phases of the experiment, following the procedure described by Tran, et al. [32]. One hour after inoculation, individual feeding of shrimp with the respective experimental diets was initiated, and it was carried out until the end of the trial (216 h post inoculation). Shrimp mortality was recorded twice a day during the challenge.

For the WSSV challenge, the WSSV Thai-1 strain [33] was used. A virus stock is kept frozen at −70 °C at IMAQUA facilities. This strain was previously isolated in Thailand from naturally-infected *Penaeus monodon* and passaged once in crayfish *Pacifastacus leniusculus* [34]. The infectivity titer of the viral suspension was determined according to a previous study [33], and the suspension was used to infect shrimp intramuscularly. The resulting infected carcasses were used to prepare the solid WSSV inoculum, which were used in the oral infection challenge trial following the procedure described by Van Thuong, et al. [35]. Each challenge trial was performed for seven days and mortality recorded twice a day. This procedure was specially designed to maximize the interaction of the experimental diets with the disease challenge and ensure that the experimental products (or their effects) were present in the shrimp’s organism at the highest level possible during infection. The clinical outcome was evaluated based on the following parameters: (i) cumulative mortality by day 7, (ii) onset of mortality, (iii) cessation of mortality, and (iv) median lethal time.

The AHPND challenge was finalized 9 days post inoculation when the control diet did not show any additional mortality in at least 24 h. The WSSV challenge was stopped 7 days post inoculation. In addition, it was ensured that the control group was without mortality event for 24 h.

### 2.6. Data Analyses

Weight gain, FCR, SGR, and survival data were analyzed using one-way analysis of variance (ANOVA), prior to which data were tested for normality and equality of group variances using the Shapiro–Wilk test and Brown–Forsythe test respectively. The Tukey’s HSD test was used when main effects were observed (*p* < 0.05). All data expressed as percentages were subjected to arcsin square root transformation before the ANOVA. For the osmotic stress test and for both disease challenge trials, survival curves were compared among experimental treatments using the Log-rank (Mantel–Cox) test, the data was also subject to Bonferroni’s correction and Chi^2^ analysis. Statistical significance was tested at the 0.05 probability level. All statistical tests were performed using Prism 6 (Graphpad).

## 3. Results

### 3.1. Growth Performance

An improved growth performance was obtained when the BSFI was introduced in the diets, regardless of the inclusion level tested (Table 2). Shrimp fed the BSFI feeds showed a significant increase in WG when compared to control shrimp (*p* = 0.0006, *F*_(3, 8)_ = 18.41, df = 3). The SGR was also lowest in shrimp fed the control diet, while those fed diets containing BSFI showed significantly higher SGR values (*p* = 0.0003, *F*_(3, 8)_ = 23.5, df = 3). Shrimp SGR significantly improved with increasing dietary BSFI inclusion, up to a 25.29% increase (BSFI10.5) compared to control shrimp. The FCR was significantly higher in the control than in the BSFI7.5 and BSFI10.5 treatments (Table 2). Survival was the only parameter that did not significantly changed with BSFI inclusion (*p* = 0.8646, *F*_(3, 8)_ = 0.2422, df = 3).

### 3.2. Salinity and Disease Challenge Tests

Introducing the BSFI showed no significant effect on shrimp survival when exposed to salinity stress (χ^2^ = 5.282, df = 3, *p* = 0.15). Shrimp fed BSFI10.5 showed a trend towards higher survival than those fed the control diet, although this difference was not statistically significant after Bonferroni correction (Table 3).

**Figure 1:**
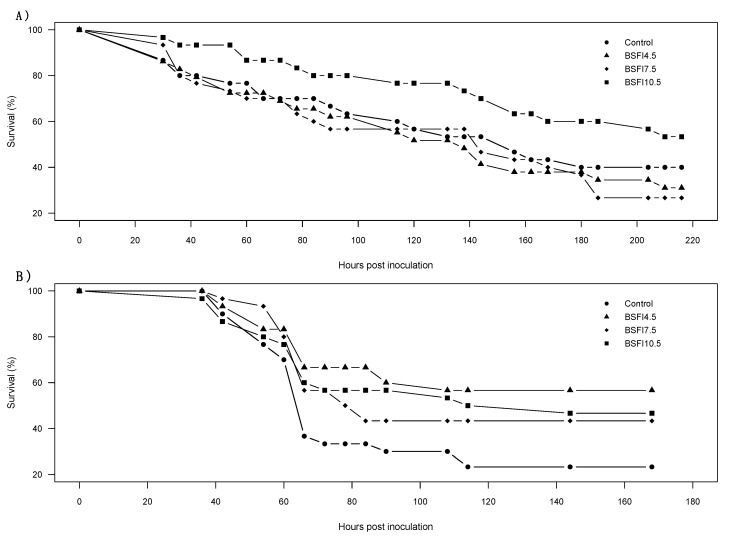
Survival curves of the acute hepatopancreatic necrosis disease (AHPND) (**A**) and white spot syndrome virus (WSSV) (**B**) disease challenge trials of *Litopenaeus vannamei* fed the four experimental diets (Control, and experimental diets with 4.5, 7.5 and 10.5% functional ingredient derived from *H. illucens* (BSFI), respectively BSFI4.5, BSFI7.5, and BSFI10.5). Survival curves were statistically similar among dietary treatments (AHPND *p* = 0.15, WSSV *p* = 0.07).

When challenged with AHPND or WSSV, shrimp survival was similar among dietary treatments (AHPND: χ^2^ = 5.382, df = 3, *p* = 0.15; WSSV: χ^2^ = 7.187, df = 3, *p* = 0.07) Figure 1. Nevertheless, a pattern of high survival for the BSFI-fed shrimp compared to the control treatment was recorded during the WSSV challenge trial (Table 3).

## 4. Discussion

Research on incorporating insect ingredients in shrimp feeds is still relatively recent [14,16,36,37]. By using BSFI inclusion rates between 4.5% and 10.5%, the present study showed that introducing *H. illucens* larvae derived products can improve the growth performance and feed conversion of juvenile Pacific white shrimp (Table 2). Similar performance improvements have been reported for Pacific white shrimp fed insect derived ingredient diets from mealworm [14,36], and for fish species fed diets containing insect ingredients from both mealworm or *H. illucens* [38,39,40,41]. In literature, such positive results have been associated with adequate amino acid profile of insect ingredients to meet aquatic species nutritional requirement [36,37,42,43]. A study performed by Barraso et al. (2014) found that Black soldier fly larvae meals have the amino acid profile most similar to fishmeal across a number of insect species which might explain the particularly good results observed. The chitin inclusion within the *H. illucens* BSFI could also potentially explain aspects of performance improvement [19], with functional effects of chitooligosaccharide (a metabolite of chitosan) shown, modulating immune-related gene expression and gut histology, boosting the performance of *L. vannamei* fed a low fish meal diet and alleviating the negative impacts of fish meal replacement [44].

In the current study, weight gain, SGR, and FCR improvement in the shrimp fed insect-based diets increased with the level of BSFI inclusion in the diet. The only other study to assess the use of a *H. illucens* derived ingredient in shrimp feeds by Cummins, Rawles, Thompson, Velasquez, Kobayashi, Hager, and Webster [16] tested the inclusion of *H. illucens* derived meal from 7% to 36% of the diet. All the test diets showed lower performance except at 7% inclusion (the only dietary inclusion level that overlaps with the present study), at which– increased WG, increased SGR, and a decreased FCR trends were observed. The comparison of these results could indicate that there exists an optimal inclusion rate that can be achieved with proportionally increasing benefits between BSFI inclusion and performance results up to this optimal rate and decreasing benefits beyond that point. The current work also indicates that there could be a potential age effect, whereby a particular sensitivity to benefits provided by the BSFI is shown in the juvenile phases of *L. vannamei.* Whilst the number of replicates is not high the growth performance data indicates that this generated considerable statistical power for the current study, and highly significant differences could be demonstrated between treatments in SGR and FCR. It can also be pointed out that the duration of the trial (28 days) was short and the study focused only on juveniles. However, when L. vannamei shrimp are around 0.1 to 0.8 g, they are in a steep part of their growth curve. In the 28-day period shrimp showed a 700% growth/7-fold size increase, which is considered by the authors enough to obtain a representative measurement of FCR and SGR, and measure differences between treatments.

Salinity changes are known to affect shrimp health and growth performance [45,46]. Although Pacific white shrimp has wide salinity tolerance (from 1 to 50 ppt), acute salinity changes lead to salinity stress and this may negatively affect shrimp health status and disease resistance, particularly to WSSV infection [47]. In the present study, *L. vannamei* resistance to acute salinity stress was not significantly affected by the introduction of the BSFI.

Research on alternative ingredient sources for aquafeeds not only aims at improving growth performance but also at improving the immunity of aquatic animals and their resistance to disease outbreaks. Choi et al. [36] and Motte et al. [14] also challenged *L. vannamei* with WSSV and *V. parahaemolyticus*, respectively, and reported that shrimp fed with insect meals showed improved survival and reduced immunosuppression compared to shrimp fed a control diet. The present study focused on challenging *L. vannamei* fed the different experimental diets with the causative agents of AHPND and WSSV, which are two of the most common diseases affecting shrimp production worldwide [26,27]. Similar to the salinity stress experiment, survival recorded for *L. vannamei* challenged with the pathogenic bacteria *V. parahaemolyticus*, the causative agent of AHPND (Table 3), were not significantly different among shrimp fed the BSFI or fish meal diets. However, the BSFI-based diets showed a tendency of increased survival when challenged with WSSV, although there was an absence of statistical significance. In order to confirm the positive trends observed of increased survival when challenged with WSSV, future trials should increase the sizes of datasets, to improve the ability to infer statistical significance. Whilst caution is recommended in making conclusions on the protective effect of *H. illucens* meals and their derivatives against WSSV, results showed a pattern for increased juvenile shrimp survival when challenged with this major pathogen. These trends might be due to the potential stimulation of the innate immune system exerted by insect chitin, or due to the lauric acid present at high concentration (40–45%) in the residual fat present in insect meal which has been shown to have anti-microbial properties [48]. In addition, there have also been more than 150 insect proteins identified as having anti-microbial properties [49]. Anti-microbial peptides (AMPs) are a broad family of peptides found widely in nature [50] and are an essential part of the innate immune response. These molecules have been shown to boost the performance of livestock animals [24,51] and shrimp more specifically [52], and indeed recently evidence of AMPs restricting infection of WSSV was shown in *L. vannamei* [53].

In addition to these existing elements, it would be interesting to explore, in future, further analyses that could allow for a greater understanding of BSFI’s potential functionality. Re-isolating the pathogens from shrimp used in the disease challenge could confirm if the potential reduction in mortality is due to the challenge diseases which would provide a clearer vision of the dataset rigor. Histopathology and lesion severity grading would provide additional information on resistance and resilience. Resistance is a reduction of the pathogen’s ability to infect the host, whilst resilience to the pathogen refers to combatting an infection to reduce its impact. Further work could explore the relative effect of the pathogens between the trial groups—gene expression related to immunology, phagocytic activity, and respiratory burst are each tools for exploring both the level of effect but also the pathways of effect.

## Figures and Tables

**Table 1:** Formulation and proximate composition of the experimental diets (Control, and test diets introducing 4.5, 7.5, or 10.5% of BSFI respectively–BSFI4.5, BSFI7.5, and BSFI10.5).

Raw Materials	Dietary Treatments			
	Control (g/100 g)	BSFI4.5 (g/100 g)	BSFI7.5 (g/100 g)	BSFI10.5 (g/100 g)
Black Soldier Fly Ingredient ^a^	0.00	4.50	7.50	10.50
Wheat, flour	35.25	32.74	31.74	31.59
Soybean meal ^b^	30.50	32.50	33.25	33.50
Fish meal ^c^	15.00	10.50	7.50	4.50
Poultry by-product meal ^d^	7.00	7.00	7.00	7.00
Sepiolite ^e^	1.50	1.49	1.21	0.60
Fish oil ^f^	2.84	2.90	2.84	2.96
Squid meal ^g^	2.50	2.50	2.65	2.65
Soy lecithin ^h^	1.50	1.57	1.67	1.78
Limestone ^i^	0.60	0.60	0.70	0.80
Vit min premix ^j^	0.50	0.50	0.50	0.50
Monocalcium phosphate ^k^	0.65	0.97	1.17	1.30
Cholesterol, feed grade ^l^	0.10	0.10	0.10	0.10
Methionine ^m^	0.05	0.12	0.16	0.21
Gelatine ^n^	2.00	2.00	2.00	2.00
Astaxanthin premix ^o^	0.01	0.01	0.01	0.01
Total	100.00	100.00	100.00	100.00
Parameter	Proximate Analysis			
DM%	100	100	100	100
Ash%	7.00	7.21	7.42	7.53
CP%	40.40	40.30	40.10	39.70
Lipid%	9.63	9.72	9.74	9.96
Fiber%	1.82	1.86	1.87	1.88
LOA (18:2n-6)%	2.03	2.08	2.15	2.23
LNA (18:3n3)%	0.16	0.16	0.17	0.17
ARA (20:4n-6)%	0.08	0.08	0.07	0.07
EPA (20:5n-3)%	0.36	0.33	0.30	0.28
DHA (22:6n-3)%	0.95	0.94	0.90	0.92
Total n-3%	1.47	1.43	1.37	1.37
Total n-6%	2.10	2.15	2.22	2.30
n-3:n-6	0.79	0.75	0.69	0.67
Total phospholipid%	2.56	2.51	2.51	2.51
Cholesterol%	0.18	0.19	0.19	0.19
Astaxanthin (mg/kg)	9.03	9.00	8.99	8.98
Arginine%	2.60	2.52	2.47	2.40
Histidine%	1.03	1.03	1.02	1.01
Isoleucine%	1.82	1.77	1.73	1.68
Leucine%	3.05	2.97	2.91	2.82
Lysine%	2.70	2.57	2.48	2.36
Methionine%	0.92	0.91	0.90	0.90
Phenylalanine%	1.88	1.85	1.82	1.78
Threonine%	1.63	1.59	1.56	1.52
Tryptophan%	0.50	0.63	0.71	0.79
Valine%	2.07	2.04	2.01	1.96
Ca%	1.49	1.42	1.42	1.40
Available P%	1.11	1.12	1.12	1.11

^a^ InnovaFeed; ^b^ 48% CP 200mn, (VDS); ^c^ 70% CP, Norvik low temperature (SEah International); ^d^ low ash, 62% CP (Cagemax); ^e^ <100 mesh, (Poortershaven); ^f^ Tuna oil, crude 031508/01 (VDS); ^g^ (Seah International); ^h^ Bergapur de-oiled, (Speerstra); ^i^ Calcium Carbonate S 48/49, (VDS); ^j^ Crustocean TV21/10, (VDS); ^k^ (VDS); ^l^ XG, (Dishan); ^m^ MERA Met Ca, (NOVUS) ^n^ (VDS); ^o^ (VDS).

**Table 2:** Growth performance of *Litopenaeus vannamei* fed the four experimental diets with different fishmeal replacement levels over 28 days. Values are presented as means ± standard deviations (different superscript letters indicate significant differences (*p* < 0.05) between experimental treatments. BSFI4.5, BSFI7.5, and BSFI10.5 indicate 30, 50, or 70% of fishmeal replacement with the BSFI, respectively).

Dietary Treatment	Final Body Weight (G)	Weight Gain (G)	Feed Conversion Ratio	Specific Growth Rate (%/Day)	Survival (%)
Control	0.52 ± 0.05	0.42 ± 0.05 ^a^	1.70 ± 0.17 ^a^	6.01 ± 0.33 ^a^	78.7 ± 14.6
BSFI4.5	0.66 ± 0.03	0.57 ± 0.03 ^b^	1.42 ± 0.07 ^a,b^	6.83 ± 0.14 ^b^	80.6 ± 2.3
BSFI7.5	0.71 ± 0.01	0.61 ± 0.01 ^b,c^	1.31 ± 0.10 ^b^	7.08 ± 0.22 ^b,c^	80 ± 8.1
BSFI10.5	0.80 ± 0.03	0.70 ± 0.03 ^c^	1.23 ± 0.12 ^b^	7.53 ± 0.18 ^c^	84.5 ± 4.3
*p* value	-	0.0006	0.0067	0.0003	0.8646

**Table 3:** Survival of *Litopenaeus vannamei* exposed to osmotic stress, acute hepatopancreatic necrosis disease (AHPND), or white spot syndrome virus (WSSV). Shrimp were fed four experimental diets over 28 days (control, and experimental diets with 4.5%, 7.5%, and 10.5% Black soldier fly derived novel feed ingredient, respectively BSFI4.5, BSFI7.5, and BSFI10.5), survival is presented as mean ± standard deviation.

	Osmotic Stress	AHPND Challenge	WSSV Challenge
Dietary Treatment	Final Survival (%)	Final Survival (%)	Final Survival (%)
Mock	0.0 ± 0.0	0 ± 0	0 ± 0
Control	33.3 ± 5.8	40.0 ± 20.0	23.3 ± 23.1
BSFI4.5	20.0 ± 10.0	31.1 ± 1.9	56.7 ± 15.3
BSFI7.5	26.7 ± 15.3	26.7 ± 15.3	43.3 ± 15.3
BSFI10.5	16.7 ± 20.8	53.3 ± 15.3	46.7 ± 11.5
*p* value	0.1523	0.1458	0.0662

## Data Availability

Data is contained within the article, represented graphically and in the form of averages with standard deviations.
